# Modeling Myxofibrosarcoma: Where Do We Stand and What Is Missing?

**DOI:** 10.3390/cancers15215132

**Published:** 2023-10-25

**Authors:** Enrico Lucarelli, Alessandro De Vita, Chiara Bellotti, Tommaso Frisoni, Silvia Vanni, Ania Naila Guerrieri, Micaela Pannella, Laura Mercatali, Marco Gambarotti, Serena Duchi, Giacomo Miserocchi, Margherita Maioli, Chiara Liverani, Toni Ibrahim

**Affiliations:** 1 Osteoncology, Bone and Soft Tissue Sarcomas and Innovative Therapies Unit, IRCCS Istituto Ortopedico Rizzoli, 40136 Bologna, Italy; enrico.lucarelli@ior.it (E.L.); anianaila.guerrieri@ior.it (A.N.G.); micaela.pannella@ior.it (M.P.); laura.mercatali@ior.it (L.M.); toni.ibrahim@ior.it (T.I.); 2 Preclinic and Osteoncology Unit, Biosciences Laboratory, IRCCS Istituto Romagnolo per lo Studio dei Tumori (IRST) “Dino Amadori”, 47014 Meldola, Italy; alessandro.devita@irst.emr.it (A.D.V.); silvia.vanni@irst.emr.it (S.V.); giacomo.miserocchi@irst.emr.it (G.M.); chiara.liverani@irst.emr.it (C.L.); 3 Unit of 3rd Orthopaedic and Traumatologic Clinic Prevalently Oncologic, IRCCS Istituto Ortopedico Rizzoli, 40136 Bologna, Italy; tommaso.frisoni@ior.it; 4 Department of Pathology, IRCCS Istituto Ortopedico Rizzoli, 40136 Bologna, Italy; marco.gambarotti@ior.it (M.G.); margherita.maioli@ior.it (M.M.); 5 Department of Surgery-ACMD, St. Vincent’s Hospital Melbourne, University of Melbourne, Melbourne, VIC 3065, Australia; serena.duchi@unimelb.edu.au

**Keywords:** musculoskeletal tissues, soft-tissue tumors, sarcoma, patient-derived cell lines, myxofibrosarcoma, disease models, cell lines, tumor models, rare tumors, in vitro, in vivo and ex vivo models

## Abstract

**Simple Summary:**

Myxofibrosarcoma (MFS) is one of the most common malignant soft tissue sarcomas. MFS occurs mostly in the extremities of patients after the fifth decade of life as a painless a slow-growing mass. In most of the cases treatment is surgical resection of the tumor. However, at times surgeons can’t distinguish the boundaries of the tumor and are unable to remove all the tumor cells. Therefore, the tumor cells left in the patients can spread and grow again (recurrence). When MFS recurs more than once it is a challenge for clinicians and a burden for patients. Therefore, especially for patients that recur more than once new therapeutic approaches are needed. In vitro and in vivo models are helpful to understand the disease and to test new therapeutic agents. This review details the available MFS models, identifies critical issues of each model, and suggests models that would be useful to develop in the future.

**Abstract:**

Myxofibrosarcoma (MFS) is a malignant soft tissue sarcoma (STS) that originates in the body’s connective tissues. It is characterized by the presence of myxoid (gel-like) and fibrous components and typically affects patients after the fifth decade of life. Considering the ongoing trend of increasing lifespans across many nations, MFS is likely to become the most common musculoskeletal sarcoma in the future. Although MFS patients have a lower risk of developing distant metastases compared with other STS cases, MFS is characterized by a high frequency of local recurrence. Notably, in 40–60% of the patients where the tumor recurs, it does so multiple times. Consequently, patients may undergo multiple local surgeries, removing the risk of potential amputation. Furthermore, because the tumor relapses generally have a higher grade, they exhibit a decreased response to radio and chemotherapy and an increased tendency to form metastases. Thus, a better understanding of MFS is required, and improved therapeutic options must be developed. Historically, preclinical models for other types of tumors have been instrumental in obtaining a better understanding of tumor development and in testing new therapeutic approaches. However, few MFS models are currently available. In this review, we will describe the MFS models available and will provide insights into the advantages and constraints of each model.

## 1. Introduction

### 1.1. Clinical Description

Myxofibrosarcoma (MFS) was originally considered a subtype of malignant fibrous histiocytoma [1], and since 2002, it has been recognized as a distinct nosological entity [2]. MFS is more common after the fifth decade of life and arises mostly in the extremities as a painless, slow-growing mass [3]. MFS may also occur in the trunk and in the neck and head region [4,5], while occurrence in the abdominal cavity and in the retroperitoneum is a rare event [6]. MFS can occur at a superficial or deep location, being the first more common [7].

MFS has several peculiar features. The tumor is lobulated and heterogeneous [8]. There is variability in cellular content, with a tendency to become more cellularized and of higher grade over time as a result of local recurrence [9]. MFS lacks a characteristic immunohistochemical expression profile. The tumor is formed by pleomorphic spindle or stellated cells within a gelatinous myxoid matrix [1,3,10]. Specific cytogenetic alterations have not been found so far, but MFS exhibits complex structural and numerical aberrations indicative of genetic instability developed in a multistep tumor progression [3,11,12,13,14]. Furthermore, the same patient can have intratumoral mutational heterogeneity [8]. In an integrated genetic and epigenetic characterization of a large cohort of MFS samples, the authors identified alteration of p53 signaling and of the cell cycle checkpoint genes in almost half of the cases, as well as relevant clusters of methylation and hypermethylation [15]. Histopathologically, MFS is characterized by other major features, particularly distinctive curvilinear thin-walled vessels [1,16].

In patients with localized disease, primary treatment is represented by surgical resection of the tumor, which can be combined with radiotherapy delivered either in the neoadjuvant or adjuvant setting. In some centers, chemotherapy is offered [17,18,19,20,21]. MFS has a peculiar infiltrative pattern, especially in superficial forms, which tend to grow into the contiguous soft tissues along normal planes, such as the fascial planes and vascular planes at microscopic and macroscopic levels [22,23,24,25,26]. Interestingly, the analysis of the 50 MFS cases at a single institution revealed that 43% of the cases had positive margins and showed microscopic as far as 29 mm from the macroscopic tumor [27]. Because of the propensity to infiltrate surrounding tissues, MFS is surgically removed with wide-margin resections. Such surgeries create large defects that require reconstruction surgery using free or rotational flaps and skin grafts. Despite this radical surgical approach, in 60% of patients, a negative margin is not obtained, and the tumor usually recurs [18,20,28]. The rate of local recurrence (LR) is particularly high compared to other STS [17,19,29].

In 40–60% of the patients, the tumor recurs more than once [29]; therefore, the patient is subjected to several local surgeries [29,30], with great discomfort for the patients and an increased cost for the health system [29]. Furthermore, each time the tumor recurs, it can show an increase in the histological grade [3,22,31], leading to decreased response to radio- and chemotherapy. This effect also contributes to a higher tendency to form metastases [24,28,29,32]. Additionally, these metastases further impact on overall survival [33].

In the case of positive surgical margins, radiotherapy is frequently used as adjuvant therapy [34,35,36], even though its beneficial effect on local tumor control is still controversial [36]. Typical schemes for intralesional surgical margins include high doses of 64–66 Gy, 2 Gy/fraction, or 45 Gy with hyperfractionation. However, MFS cells frequently survive radiotherapy, and the tumor can recur in spite of the treatment [19,36,37]. However, in recent years, preoperative hypofractionated radiotherapy (HFRT) has emerged as a reasonable treatment modality, showing favorable oncologic outcomes and toxicity profiles compared to normofractionated RT. Indeed, more than 15 phase II clinical trials applying HFRT with or without chemotherapy in STS are currently ongoing [38], suggesting that shortening RT courses could improve therapy adherence, increase cost-effectiveness, and ultimately provide additional treatment options for a wider range of patients.

Moreover, as already reported, chemotherapy could be an option for the treatment of localized disease, and it represents a cornerstone in metastatic MFS. In this regard, the role of chemotherapy is still debated, and the achieved outcomes are very poor. The limited available data on the role of chemotherapy in MFS is due to the few cohort studies conducted. The standard first-line chemotherapy is represented by an anthracycline-based regimen. These studies suggest that overall survival is not significantly increased after chemotherapy [17,20]. No general consensus has been reached yet for second-line medical treatment, which is generally represented by ifosfamide, pazopanib, and gemcitabine-based chemotherapy and often combined with docetaxel, with a response rate of 10% [39,40,41]. Anectodical studies have reported the use of temozolomide followed by atezolizumab [42] and the combination of nivolumab and bevacizumab [40,43,44], with limited evidence due to only a single investigated case.

Potential prognostic predictor factors are grade, appropriate margins, primary unplanned resection at another facility, age, size, and depth [18,20,30,36,45]. The overall 5-year survival rate ranges between 60 and 70% [17,20,29,31,33,39]. Thus, for MFS patients, the current therapeutic options are inadequate, and there is a constant effort to develop innovative therapeutic strategies [45,46].

From a clinical standpoint, there are two major challenges with MFS patients: the first one is to decrease the rate of local recurrence, and the second one is the identification of an effective adjuvant or neoadjuvant therapy to control the disease in patients in aggressive or metastatic settings. In both cases, preclinical models can be extremely useful to identify innovative therapeutic strategies.

### 1.2. Modeling Myxofibrosarcoma: Where Do We Stand?

Modeling MFS involves careful consideration of various factors to create accurate and meaningful representations of the disease. Here are some important aspects to consider but do not necessarily reflect what has been achieved so far:

1. Biological Complexity: MFS exhibits intricate biological behavior, including tumor heterogeneity, microenvironment interactions, and genetic variability. Models should aim to capture this complexity to provide insights that mirror real-world scenarios.
2. Histopathological Features: MFS has distinct histopathological features. Models should incorporate these features, such as the presence of myxoid and fibrous components, to accurately simulate the tumor’s appearance and behavior.
3. Genetic and Molecular Characteristics: Understanding the genetic and molecular underpinnings of MFS is crucial. Models should integrate genomic data to simulate the mutations and molecular pathways driving tumor growth and progression.
4. Tumor Microenvironment: It is important to consider the interactions between tumor cells and their microenvironment, including immune cells, blood vessels, and extracellular matrix components. Modeling the tumor microenvironment can shed light on immune responses and potential therapeutic targets.
5. Invasion and Metastasis: MFS is known for its infiltrative behavior and potential for metastasis. Models should replicate these aspects to understand how the tumor invades surrounding tissues and spreads to distant sites.
6. Long-Term Behavior and Recurrence: It is important to incorporate dynamics that account for tumor growth over time, potential recurrence, and regrowth after treatment. Long-term modeling can help assess treatment strategies and disease management.
7. Clinical Data Integration: Utilizing clinical data to validate and refine models is essential. Patient data, such as treatment responses, outcomes, and demographic information, can enhance the relevance of the simulations.
8. Therapeutic Interventions: This involves incorporating various treatment modalities, including surgery, radiation, and chemotherapy. Models can help predict the efficacy of different treatments and optimize therapeutic strategies.
9. Patient-Specific Modeling: This entails exploring ways to personalize models using individual patient data. Patient-specific modeling can guide treatment decisions and predict outcomes based on a patient’s unique characteristics.
10. Validation and Benchmarking: Developing protocols for validating and benchmarking the accuracy of models can be used to compare model predictions with clinical observations and experimental data to ensure reliability.
11. Interdisciplinary Collaboration: Modeling MFS requires collaboration among researchers with diverse expertise, including oncology, pathology, biology, mathematics, physics, and computational sciences. A multidisciplinary approach ensures a comprehensive understanding of the disease.
12. Ethical Considerations: It is important to acknowledge ethical concerns related to patient data usage, model transparency, and potential clinical applications and ensure that the models adhere to ethical standards and guidelines.
13. Model Interpretability: This involves striving for models that are interpretable and can provide actionable insights for clinicians and researchers. Transparent models can help bridge the gap between computational predictions and clinical decision-making.

**Figure 1:**
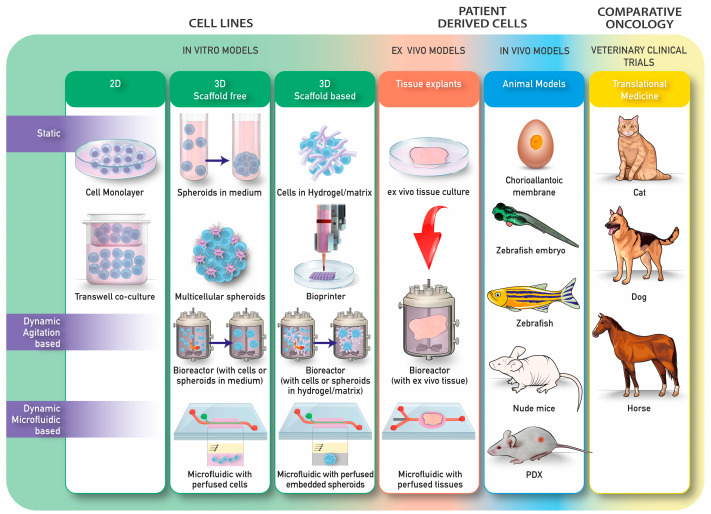
Overview of the preclinical models that are used for research in other tumors and might be relevant for MFS research. The in vitro models can be divided in 2D and 3D models.

For other tumor types, there is a large array of models, both in vitro and in vivo, available today. The in vitro models span from simple 2D cultures with a single cell type to complex 3D co-culture systems. The in vivo models include preclinical models and comparative medicine (Figure 1). This paper will provide a comprehensive overview of MFS models that have been obtained so far.

## 2. Available MFS Models

### 2.1. MFS In Vitro Cell Cultures

Over the last 7 decades, cells grown in Petri dishes in monolayer (2D cultures) have been a basic tool used to investigate molecular mechanisms of cancer progression, to identify receptors and/or cell surface molecules, and to study the effect of anticancer drugs at the cellular and subcellular levels.

#### 2.1.1. Immortalized MFS Cells Grown in Monolayer Cultures

For all types of tumors, primary cells are the primary preclinical tool. In the case of MFS, to the best of our knowledge, 35 different immortalized MFS cell lines have been published (Table 1), and several short-term MFS cell cultures have been described (Table 2). In our list, we have also included lines that were described before MFS became a distinct entity in 2002; in these papers, MFS cells are defined as malignant fibrous histiocytoma (MFH) with a confirmed myxoid subtype. For example, the isolation of the first cell lines that can be reconducted to the current definition of MFS was published in 1992 [47]. In this paper, the authors isolated several MFH lines from biopsies taken from patients who underwent surgery at their hospital. From three patients with the myxoid subtype of MFH, they obtained three lines: SFT85-06, SFT79-08, and SFT81-12.

Overall, all of the 35 immortalized MFS cell lines published retain the characteristics of the tumor of origin. For example, cells in culture resemble the cells in the histological sections, with the spindle-shaped phenotype. In some papers, a subpopulation of giant cells, that is, a fraction of the spindle cells, has been identified [47]. In general, these giant cells are detectable in the initial stage of culture and persist through the passages [13,48,50,52,53,54,57], while in another article, the giant cell population was gradually overgrown by spindle cells [49]. On the nature of these gigantic cells, Ariizumi et al. demonstrated that they may originate from uncompleted cell division of the MFS spindle-shaped cells [52]. This population of bizarre gigantic cells is also found in other subtypes of MFH [47,70] and might represent an important common feature between MFH and MFS. It is not clear whether the presence of these gigantic cells in culture is patient-dependent or whether it depends on the culture conditions, such as the presence of a cell culture media that can enable the survival of a determined cell population. Unfortunately, the protocols for isolation and culture of the cells are not uniform among the groups, so it is difficult to discriminate whether the absence of the second phenotype of cells in some of the cultures depends on the protocol of isolation, the media, or if it only happens in particular culture conditions. To be able to compare results among laboratories, it is best to identify standard culture conditions to be shared among researchers worldwide. This will enable the comparison of results on MFS cell lines between laboratories. We believe that this effort should be made while few MFS cells are available and while few laboratories are involved in MFS tissue culture.

Although MFS cell lines resemble the cells of the original tumor from a genetic perspective, no specific cytogenetic alterations have been identified so far. In fact, MFS cells in patients are characterized by complex cytogenetic aberrations such as polyploidy, extra copies and amplification of chromosome regions, ring chromosomes, gene fusions, and telomeric association. MFS cell lines in vitro have alterations of the karyotype, such as heteroploidy [50], and the number of chromosomes is often variable. For example, the NMFH-1 cell line has more than 84 chromosomes [50], the MFS line Nara-h has 109 chromosomes [49], the OH931 cell line has subclones with more than 120 chromosomes [48], and the Shef-MFS 01 cell line has 120 chromosomes as well [54]. In addition, MFS cells are characterized by elevated genetic instability. Interestingly, Lohberger et al. isolated two subclones derived from the same tumor specimen, namely MUG-Myx2a and MUG-Myx2b cells, that share mutations in FGF3, KIT, KDR, and T53 genes, while the PTEN gene is mutated in MUG-Myx2b cells only [13]. Li et al. developed five MFS cell lines that have variable levels of deletion in RB1 and TP53. In particular, one cell line has both missense mutations and shallow copy deletion in RB1 [60]. Furthermore, the cell line NCC-MFS3-C1 has multiple chromosome deletions [63].

MFS cell lines are characterized by persistent and uncontrolled proliferation, and in all the papers, the cell cultures were stable for months. However, it is possible that MFS cells can acquire extensive genetic alterations while in culture because of long-term passaging. This stability may imply that the genetic variation of the cells is artificially worsened, as it is also for other tumor cell types [71]. Therefore, it should be recommended to conduct experiments when the passage of the culture is minimal, ensuring that cells are more representative of the original tumor.

Another key characteristic of MFS is the ability to infiltrate surrounding tissues for several centimeters as well as at the microscopic level. MFS often show abnormal curvilinear infiltration along the fascial plan; these extensions are generally indicated as tails [22,23,24,25,26]. Despite being such an important feature of MFS, currently, we know very little about the migratory properties of MFS cell lines. Only a few papers have published results on the migration and invasion properties of MFS cells [13,55,59,63,65,66,72]. In some of them, the authors demonstrated that NCC-MFS-1-C1 and NCC-MFS2-C1 are able to invade the matrix using Matrigel invasion chambers [58,62]; moreover, Li et al. showed that bortezomib inhibits the invasion potential of NMFH-1 cells [73]. It has also been demonstrated that the lines NCC-MFS4-C1 and NCC-MFS6-C1 are more invasive than the osteosarcoma MG63 cells [64,66]. In addition, Okada et al. showed that the overexpression of integrin-A10 in MFS cells leads to an enhancement in cell migration and invasion in vitro [55]. In a recent paper, five MFS cell lines were obtained from different patients, and their invasive properties were compared. The results show that all MFS cell lines have good invasive potential, even with a variable extent [65]. Invasiveness is a key feature of MFS cells, and it is at the base of the recurrence and metastasis of this tumor. Thus, we believe it deserves to be further investigated.

Monolayer MFS cultures are mostly used as an early screening system to study the sensitivity of the cells to new drugs. MFS is considered refractory to chemotherapy, for which the treatment of patients using a combination of anthracyclines and ifosfamide is generally only marginally effective [17,39].

MFS cell lines have been proven to be sensitive to classical drugs such as trabectidin, epirubicin, cisplatin, and doxorubicin [13,51,57,59,74]. Several classes of drugs have been shown to be effective in monolayer cultures. In a few high-throughput studies, MFS cells were exposed to hundreds of agents; for example, NCC-MFS-1-C1 was found to respond to ponatinib, vandetanib, bortezomib, and romidepsin [58]. High-throughput studies were also conducted by Professor Kondo’s group using MFS cells. The so-obtained results show that many of the analyzed drugs do not affect the proliferation of MFS cell lines. Furthermore, these cell lines have different sensitivities to tested drugs [62,63,65,66]. These studies confirm that MFS cells only respond to a limited number of drugs in vitro and are resistant to many others.

Altogether, these results highlight the importance of immortalized MFS cells grown in monolayer cultures in preliminary drug screening, and promising drugs should be further tested in more complex preclinical models to speed up the translation of these results toward the clinical application and to increase the spectrum of therapeutic agents that can be used to control MFS growth.

Interestingly, the efficacy of MFS can be dependent on many factors, among them the genetic background of the cells. Different clones of immortalized cells obtained from the same biopsy can represent intratumoral heterogeneity. For example, Lohberger et al. exposed two distinct cellular clones—namely MUG-Myx2a and MUG-Myx2b—obtained from the same donor specimen to doxorubicin, verinostat, and borezomib and found that all three drugs inhibited cell growth in a dose-dependent manner, even if there was a significant difference in inhibition among the two clones [13]. Similar results were obtained when MUG-Myx2a and MUG-Myx2b were exposed to periplocin [61]. Differences among clones were also found by Li et al., who investigated, in 2D culture conditions, whether ALDH1 expression in MFS cells influences cancer stem cell features. NMFH-1 cells were exposed to increasing concentrations of doxorubicin and cisplatin, and the results demonstrate that ALDH1+ cells were more resistant to these drugs than ALDH1- cells [74]. Furthermore, according to Miserocchi et al., not only is the clonal population a determining factor for the sensitivity to a drug, but the passage of the cells in culture is as well. In fact, the authors showed that the sensitivity of the MFS cell line IM-MFS-1 to trabectidin and epirubicin increased with time, as the cells at passage 50 were more affected by the treatment compared to passage 1 [57]. Therefore, there is a need to include as many MFS cell lines as possible to set up a test that can be representative of the efficacy of a drug for MFS.

Monolayer cultures can also be used to identify prognostic factors. The OH931 and NMF-1 cell lines have been used to test whether MET could be used as a prognostic factor for tumor progression, invasion, and metastatic spread [75]. These authors found, by direct sequencing, that both cell lines have wild-type MET. Western blot analyses further determined that MET was highly expressed in OH931 cells while the protein expression in NMF-1 cells was lower. Once the cells were exposed to HGF, MET was phosphorylated, although to different extents, as it was more phosphorylated in OH931 than expected. Therefore, the MET pathway is active, and MET could be a potential prognostic factor.

MFS tumor masses are characterized by the presence of immune cells that probably have a specific role in neoplastic development. In fact, there could be crosstalk between different cell types that is definitively worth studying. For example, Shiraishi et al. used MFS cell monolayer cultures to study cell–cell interaction. In this paper, the cell line NMFH1 has been cocultured with CD106 expressing macrophages. The results demonstrated that macrophages induce MFS cell growth, while MFS cells induce the expression of CD106 in macrophages [76].

#### 2.1.2. Conclusions and Further Outlook for Immortalized MFS Monolayer Cultures

MFS cell cultures are representative of the MFS tumor and are a fundamental tool for research. So far, 35 distinct MFS lines have been published, but given the complexity of this tumor, there is a definitive need for more cell lines. For example, there are only a few lines that originate from the same biopsy. This is problematic because these cultures may be representative of the heterogeneity of MFS and are a fundamental tool to understand the biology of the tumor and to test therapeutic agents. Additionally, most of the available MFS cell lines originate from a primary tumor (Table 1), besides the fact that few of them originate from a recurrence and none from a metastasis. Due to the extreme complexity and heterogeneity, we need cell lines that represent different MFS grades and stages. It would also be of high value to obtain cultures from the same patient after each recurrence to follow the neoplastic progression because they may respond differently to drugs. Furthermore, since the ability to invade other tissues is a key characteristic of MFS, more attention should be paid to the cells that constitute the periphery of the tumor. As far as we know, to date, there are no studies that compare MFS cultures originating from the tails to cultures established from the central tumor mass.

Another aspect where there is room for improvement is the characterization of these MFS cells in culture. This might be a daunting task because MFS lacks specific immunohisto-chemical markers and a distinct genetic profile, but “minimal criteria” for defining MFS cells must be developed.

### 2.2. MFS Cells in Three-Dimensional MFS Cultures

Three-dimensional (3D) cultures are more suitable for recapitulating tumors than two-dimensional (2D) ones because they better recreate the physiological cellular microenvironment in vitro. There are different kinds of 3D cultures, principally divided into scaffold-free and scaffold-based ones, where polymeric biomaterials might be used to support cell adhesion and cell–cell interactions. Compared to 2D cultures, 3D cultures have a greater potential to represent the natural environment and the physiological response to drugs.

#### 2.2.1. MFS Spheroids and Sarcospheres

Spheroids are the simplest and most widely used scaffold-free 3D culture system for MFS cell lines. They consist of cellular aggregates made by forcing cells to adhere to each other and to grow in suspension. This 3D model is commonly used for many types of tumors, particularly in high-throughput drug screening. In certain cases, the cells of interest can bind with polymeric biomaterials to form a matrix in which cells create a microenvironment that highly mimics the original tumor. Spheroids have been used to study the roles of tumor cells and the microenvironment through the incorporation of varying types of cells, such as mesenchymal stem cells and osteosarcoma cells [77]. The most notable advantages of this model are its low production costs and high reproducibility. While some MFS cell lines form spheroids naturally, others need round-bottom and/or low attachment plates in order to do so [58,62,63,64,65,66].

Kenan et al. obtained spheroids composed of 5000 MUG-Myx1 cells and used them to test the effect of photodynamic therapy for MFS; in this paper, the authors demonstrated that the photosensitizer 5-ALA was internalized by MFS cells that died upon photoactivation [78]. In a previous study, Li et al. investigated in vitro whether ALDH1-expressing MFS cells have cancer stem cell characteristics using the cell line NMFH-1 isolated by Kawashima et al. [50]. After cell sorting, the authors demonstrated that the ALDH1+ cells form spheroids in a more efficient manner than NMFH-1 ALDH1- cells. Similar results from the 2D cultures were obtained when spheroids were used, demonstrating that ALDH1+ cells maintained their higher resistance to doxorubicin and cisplatin [74]. In a second paper, Deng et al. showed that spherical colonies composed of NMFH-1 cells are more resistant to doxorubicin and cisplatin compared to monolayer cultures [79].

Chen et al. isolated MFS cells from two donors and obtained USZ20-MFS-1 and USZ20-MFS-2 cells. These cells were cultured in 2D tissue flasks for up to five passages in ultra-low attachment conditions and formed round cell aggregates identified as sarcospheres. The authors characterized the sarcospheres via next-generation sequencing and methylation profiling. Sarcospheres originating from both donors were used to screen the potential efficacy of drugs alone or in combination. Both were sensitive to doxorubicin, trabectidin, PU-H71, and carfilzomib when administered separately or together. Finally, Pauli et al. used WCM197 cells isolated from surgical specimens of a female MFS patient to obtain 3D sarcospheres by plating the cells in Matrigel drops. Sarcospheres were also used for drug screening in this experiment [67].

In conclusion, these examples show the ability of MFS to grow in spheroids and that this model can be used for high-throughput drug screening and to identify new drugs to control MFS recurrence and growth.

#### 2.2.2. MFS Cells Grown on 3D Scaffolds

De Vita et al. obtained three different MFS cultures (MF1, MF2, and MF3) from three distinct patients affected by high-grade MFS. The authors investigated the efficacy of epirubicin, ifosfamide, and trabectidin when the cells were infused in a 3D collagen scaffold. Notably, there was a high degree of variability in the toxicity among the three cultures depending on whether the drugs were administered alone or in combination. Specifically, the MF2 responded less than the other cultures [56]. The response to epirubicin of MFS cultures grown in a collagen scaffold was recently tested in short-term primary cultures derived from 12 MFS patients, the results of which confirmed a high degree of variability in the response to the drugs [80].

#### 2.2.3. Conclusions and Further Outlook for MFS 3D Cultures

A few examples of MFS 3D cultures have been published thus far, all of which prove the need to transition from 2D to 3D models for these types of cells in preclinical studies. Such 3D models are particularly relevant for MFS, not only to produce more trustworthy evidence of the drug’s efficacy but also due to their fundamental contribution to the investigation of the tendency of MFS to infiltrate other tissues and migrate along the fascia. These peculiar features of MFS may be triggered by inflammatory cytokines secreted by immune cells that populate the MFS microenvironment. Therefore, 3D models of co-cultured cells may be extremely useful to understand the biology of the tumor and to develop new therapeutic strategies to reduce the recurrence rate. As progress is made in the in vitro culture of MFS and new technologies become available, such as 3D printing and microfluidic cultures, these techniques may enable us to develop more sophisticated models and tools to test the efficacy of new therapeutic approaches.

### 2.3. In Vivo MFS Models

In vivo models are primarily generated by the implant of cells expanded ex vivo in culture (cell line-derived xenograft) or by the direct implant of tissue portions derived from biopsies (heterotransplanted tumor). Both types of implants have advantages and disadvantages; the use of cultured cells allows the insertion of a predetermined number of cells in each animal, making it easier to compare results obtained within the same experimental group. Furthermore, the cell lines could be transfected with vectors that allow them to become bioluminescent or fluorescent to be easily tracked with optical imaging instrumentation such as IVIS ^(R)^. In this case of a cell line-derived xenograft, however, the contribution of the tumor microenvironment is lost, leading to a lack of consistency between the generated models and the real conditions. Contrarily, the use of neoplastic tissue fragments permits the implant of tumor cells within their natural microenvironment, creating an environment more similar to the real conditions within the patient. On the other hand, MFS are non-homogenous tumors with variable cell density and composition, making each implant unique and thus difficult to compare to other implants in the same experiment. Furthermore, the number of cells that can be isolated from a bioptic specimen is far inferior to the number of cells that can be obtained with cell lines, limiting the number of replicas that can be obtained in a single experiment.

As far as we know, the two in vivo models that have been used to study MFS are the chicken chorioallantoic membrane (CAM) and the nude mouse.

In the first case, fertilized chicken eggs between day 5 and day 6 are generally used; during this period, the fusion of the chorion and the allantoic membrane allows the formation of the chorioallantoic membrane, which creates the conditions most similar to the placenta in mammals for fetal respiration and metabolism [81]. In the CAM assay, rich vascularization of the chorioallantoic membrane supports the grafted cells. CAM is used to study angiogenesis, tumor growth, and the process of metastases development. This model is affordable, and if used before the immune system starts functioning, it has the advantage of being an immunodeficient model [82,83]. Additionally, the use of this model can spare animal discomfort and suffering since the nociception and ability to experience pain starts after the second week [84,85,86].

Several models of MFS cells implanted in mice have also been published. These models represent the different forms of MFS that occur in the patient. MFS occurs superficially in dermal and subcutaneous tissues as well as in the muscle layers, mainly in the subfascial layer [87]. In the majority of published studies with myxofibrosarcoma animal models, MFS cells are implanted under the skin of the mice, representing the subcutaneous model of superficial MFS tumors. Conversely, in other papers, the MFS cells are implanted in the muscle, representing deep MFS forms.

#### 2.3.1. Cell Line-Derived Xenograft

##### Cell Line-Derived Xenograft in CAM

In two studies, 3D cellular xenografts obtained from MFS cells (MUG-myx1 and MFS-SN1) were implanted in CAM to test the efficacy of photodynamic therapy for control of local recurrence in soft tissue tumors. In both cases, results demonstrated that photoactivation of the photosensitizer 5-aminolevulinic acid (5-ALA) greatly reduced xenograft volume [69,78].

##### Mouse Models for Superficial MFS

In most of the published articles, MFS cells have been implanted subcutaneously to test drug efficacy. This model offers several advantages; apart from being technically easier than a deeper implant, it allows us to visually assess the growth of the tumor mass, which can be measured with either a caliper or with in vivo imaging systems, according to whether deep sedation of the animal can be performed. As a result, this model has been used to obtain evidence of concepts on the efficacy of drugs in many papers, all of which have proven the model to be consistent and reliable. Tumors have been grown with an elevated percentage of success with various MFS cell lines implanted alone or embedded in Matrigel [13,49,50,52,55,63,64,65,66,67,72,73,74,88]. To our knowledge, only NCC-MFS6-C1 cells failed to form tumors when implanted in nude mice [66]. In conclusion, most of the tested MFS lines can form superficial tumors when implanted subcutaneously if engraftment occurs successfully.

##### Mouse Model for Deep MFS

As far as we know, only one report of a mouse model of deep MFS has been reported. Krause et al. implanted OH931 MFS cells in a muscle of the thigh of athymic mice and obtained a visible mass in two weeks. Histology performed at six weeks showed that the tumor had a morphology comparable to the primary tumor with pleomorphic mononuclear cells and multinucleated cells with a complex karyotype immersed in a myxoid matrix [48].

This paper contains evidence that MFS cells can also grow when implanted in deep tissues. We recommend the deep tissue injection testing of other previously successful MFS lines to further develop the study of deep MFS mouse models.

##### MFS Mouse Lung Metastasis Model

MFS metastasis generally occurs in the lungs, lymph nodes, and, in a few instances, in other locations in patients [28].

Okada et al. injected luminescent MXF8000 cells in the tail vein of NSG mice. Mice were treated with p21-activated kinase and mTOR inhibitors. Four weeks after the injection, the authors detected a reduction in the luminescence in the lung consistent with a reduction in the size of the metastases [55]. This paper thus proves that the model of lung metastasis can be established by injection of MFS cells in the tail vein.

#### 2.3.2. Heterotransplanted Tumor

##### Patient-Derived Xenograft in CAM

Recently, a large collection of sarcomas was implanted in the CAM, two of which were MFS. These experiments were performed to test the feasibility of photodynamic detection and the efficacy of photodynamic therapy of the photosensitizer aminolevulinic acid (5-ALA). The results demonstrated that MFS cells were able to incorporate the photosensitizer [89].

##### Patient-Derived Xenograft (PDX) Mouse Model

In the literature, several examples of PDX mouse models of MFS are present. For example, Cornillie et al. harvested MFS tissue from 21 patients and successfully established seven MFS PDX by transplanting MFS bioptic fragments of 25–75 mm^3^ in the subcutaneous tissue of the side of immunocompromised mice [90]. In another paper, Kiyuna et al. initially generated a subcutaneous implant of small fragments of an aggressive MFS in nude mice, and after three weeks, when the mass was over 10 mm in diameter, the re-fragments of the tumor were implanted in the biceps femoris of the mice. The mice carrying the orthotopic PDX were exposed to a combination of drugs when the tumor reached 70 mm^3^. The authors described that doxorubicin, pazopanib, and temozolomide did not affect the tumor growth, while irinotecan alone or in combination with temozolomide, or in combination with cisplatinum, decreased tumor growth. S. typhimuriom also arrested the growth of the tumor [91].

##### Conclusions and Further Outlook for MFS In Vivo Models

MFS in vivo models constitute fundamental tools to test the efficacy of therapeutic strategies to control a tumor’s growth locally. The results obtained with the photoactivation of photosensitizers already in clinical practice for other diseases can be easily translated into clinical trials.

As of now, only a few in vivo MFS models are currently available and can be used to study tumor biology and test new therapeutic options. To improve the outcome in MFS patients, we need to increase the number of models useful for preclinical studies and engineer them to have a higher degree of complexity to recapitulate the heterogeneity of MFS. For example, it would be interesting to develop ex vivo tumor models that better recapitulate the physical aspects of the MFS microenvironment and the interaction with the immune system.

Additionally, these new tools provide insight into important aspects of MFS biology and address unresolved clinical issues, ultimately improving patient outcomes. Similarly, it is recommended to overcome the limitations of the available models. For example, in PDX, the microenvironment of the original tumor is gradually replaced with the mice microenvironment during passages and may be less representative of the original tumor. In xenograft tumors based on in vitro expanded MFS cells, we can select only the clones that are more adaptable to survive the artificial in vitro environment and are not representative of the original tumor population and heterogeneity.

Furthermore, in the case of MFS, only a few of the possible models have been developed and employed. For example, to our knowledge, no zebrafish models have been developed to visualize the migration and invasion of MFS cells. The use of mice with a humanized immune system, such as mouse strain MISTRG [92], may be useful to study immune cell interaction by implanting the patient’s tumor along with monocytes derived from the patient’s peripheral blood. Also, no large animal models are available. It is our opinion that more funds should be devoted to the development of MFS animal models.

## 3. Veterinary Research

We could obtain beneficial results for both human and animal species from more direct collaboration between veterinarians and experts on human health. For example, dogs can develop tumors at a higher rate than humans; osteosarcoma (OS), a rare tumor in humans, is 75 times more frequent in large dog breeds [93]. Taking into consideration that breed dogs have fewer genetic variations, in the case of OS, dogs may represent a timely and more efficient method to study tumor development.

Companion animals may develop tumors spontaneously, which is less artificial than models in which tumor cells of human origin are implanted. The tumor that these animals develop is more representative of the tumor in human patients since the immune system is generally functionally active, so the newly formed neoplastic mass maintains its heterogeneity. Therefore, these spontaneous tumors offer the chance to experiment with new therapeutic strategies in veterinary clinical trials in an environment that is more similar to the human clinical one. An animal lifespan shorter than that of a human facilitates a faster acquisition of data and of subsequent evaluations of the efficacy of cancer treatments. The size of large dogs is also more comparable to the size of human patients, allowing the use of comparable diagnostic, surgical, and radiological instrumentations. A variety of STSs have been described in dogs, cats, and horses. STSs are more frequent in large dog breeds, and among them, some also develop MFS [93]. In particular, Milovancev et al. have described six cases of canine MFS with clinical presentation and histological features equivalent to their human counterparts [94]. As far as we know, MFS has only been found in dogs. However, it is possible that we are not aware of other companion or domestic animals that can develop MFS.

## 4. What Is Missing in Myxofibrosarcoma Modeling?

In the context of scaffold-based 3D models, since the MFS matrix is mostly composed of glycosaminoglycans, MFS cells should be cultured in 3D scaffolds, or gels, including Heparan sulfate or chondroitin sulfate [9]. In addition, as far as we know, no microfluidic studies have been performed so far with MFS cells. Advanced microfluidic models have been used to study the mechanisms of invasion of tumor cells [95]. In the case of MFS, microfluidics platforms could be particularly useful to investigate the process of MFS invasion of the contiguous soft tissues at the microscopic level, which probably is what causes MFS’s high recurrence rate. For this purpose, the zebrafish embryo could also be extremely useful in studying neoplastic invasion at the single-cell level.

## 5. Conclusions and Final Remarks

To date, only a few in vitro and in vivo preclinical MFS models are available, but each one gives us information from a unique perspective. For example, if we want to study the biology of living MFS at the single-cell level, we can revert to in vitro tests or to zebrafish embryos. This is because it is technically more difficult to study the single cell when cells are implanted in a mouse model. Furthermore, we need to consider that the complexity of the model is directly correlated to the costs and effort required for the experiments, leading to an inverse correlation with experimental throughput. For example, drug screening is generally started in less-expensive, high-throughput 2D cultures, and results obtained are later validated in more complex settings. Therefore, different models are necessary to answer different scientific questions. If these models are used in a complementary and integrated manner, they can give us a better comprehension of the biology of the tumor and the data necessary to prove the effectiveness of new therapeutic strategies. In this review, we have illustrated what models are currently available for MFS research and propose other possibilities that will have to be developed in order to better understand MFS pathobiology and be able to test new therapeutic approaches.

## Figures and Tables

**Table 1:** MSF immortalized cell lines.

#	Cell Line	Tumor Characteristics	Gender	Age at the Time of Surgery (years)	(Primary Tumor/ Local Recurrence/ Metastasis)	Cell Morphology	References
1	SFT85-06	un.	M	65	Primary tumor	Double population	Iwasaki et al., 1992 [47]
2	SFT79-08	un.	M	80	Primary tumor	Double population	Iwasaki et al., 1992 [47]
3	SFT81-12	un.	M	85	Primary tumor	Double population	Iwasaki et al., 1992 [47]
4	OH931	Deep mass in the thigh grade II and III	F	73	Primary tumor	Single population	Krause et al., 1997 [48]
5	Nara-F	Mass of the uterus	F	68	Primary tumor	Double–single population	Kiyozuka et al., 2001 [49]
6	Nara-H	Mass of the uterus	F	68	Primary tumor	Double–single population	Kiyozuka et al., 2001 [49]
7	NMFH-1	Supeficial left knee	M	89	Recurrence	Double population	Kawashima et al., 2005 [50]
8	CNIO-BG	un.	un.	un.	un.	un.	Moneo et al., 2007 [51]
9	NMFH-2	Upper left arm	M	79	Primary tumor	Double population	Ariizumi et al., 2009 [52]
10	MUG-Myx1	Thorax	M	66	Recurrence	Double population	Lohberger et al., 2013 [53]
11	Shef-MFS 01	Upper limb	M	73	un.	Single population	Salawu et al., 2016 [54]
12	Shef-MFS 02	Upper limb	M	73	un.	Single population	Salawu et al., 2016 [54]
13	MXF8500	un.	un.	un.	un.	Single population	Okada et al., 2016 [55]
14	MXF2734	un.	un.	un.	un.	Single population	Okada et al., 2016 [55]
15	MXF800	un.	un.	un.	un.	un.	Okada et al., 2016 [55]
16	MXF9100	un.	un.	un.	un.	Single population	Okada et al., 2016 [55]
17	MUG-Myx2a	G3	F	94	Primary tumor	Single population	Lohberger et al., 2017 [13]
18	MUG-Myx2b	G3	F	94	Primary tumor	Single population	Lohberger et al., 2017 [13]
19	MF-1	G3 thigh	F	66	Recurrence	Single population	De Vita et al., 2017 [56]
20	MF-2	G3 of the knee	M	69	Recurrence	Single population	De Vita et al., 2017 [56]
21	MF-3	G3 arm	M	64	Primary tumor	Single population	De Vita et al., 2017 [56]
22	IM-MFS-1	Knee	M	69	Recurrence	Single population	Miserocchi et al., 2018 [57]
23	NCC-MFS1-C1	Subcutaneous forearm	M	82	Recurrence	Single population	Kito et al., 2019 [58]
24	S57	un.	un.	un.	un.	Single population	Piano et al., 2020 [59]
25	8000s	un.	un.	un.	un.	un.	Li et al., 2020 [60]
26	8500	un.	un.	un.	un.	un.	Li et al., 2020 [60]
27	9172	un.	un.	un.	un.	un.	Li et al., 2020 [60]
28	2734	un.	un.	un.	un.	un.	Li et al., 2020 [60]
29	3672-3	un.	un.	un.	un.	un.	Li et al., 2020 [60]
30	T60	Grade 3	M	62	Primary	un.	Lohberger et al., 2020 [61]
31	NCC-MFS2-C1	un.	M	71	Primary	Single population	Noguchi et al., 2021 [62]
32	NCC-MFS3-C1	un.	F	74	Primary	Single population	Tsuchiya et al., 2021 [63]
33	NCC-MFS4-C1	un.	F	65	Recurrence	Single population	Yoshimatsu et al., 2021 [64]
34	NCC-MFS5-C1	un.	M	60	Primary	Single population	Tsuchiya et al., 2022 [65]
35	NCC-MFS6-C1	un.	F	85	Primary	Single population	Yoshimatsu et al., 2022 [66]

Gender (M = male/F = female), cell morphology (single population/double population), un. = undisclosed.

**Table 2:** Short-term MFS primary cultures.

#	Cell Line	Tumor Characteristics	Gender	Age at the Time of Surgery (years)	(Primary Tumor/Local Recurrence/Metastasis)	Cell Morphology	References
1	WCM197	un.	F	un.	Primary tumor	Single population	Pauli et al., 2022 [67]
2	USZ20-MFS1	un.	M	66	Recurrence after radiation	Double population	Chen et al., 2023 [68]
3	USZ20-MFS1	un.	M	71	Primary tumor after radiation	Single population	Chen et al., 2023 [68]
4	MFS-SN1	un.	un.	un.	un.	un.	Kerkhoff et al., 2023 [69]

Gender (M = male/F = female), cell morphology (single population/double population), un. = undisclosed.

## Data Availability

Not applicable.
